# Freestanding Activated Carbon Nanocomposite Electrodes for Capacitive Deionization of Water

**DOI:** 10.3390/polym14142891

**Published:** 2022-07-16

**Authors:** Humair Hussain, Asim Jilani, Numan Salah, Ahmed Alshahrie, Adnan Memić, Mohammad Omaish Ansari, Joydeep Dutta

**Affiliations:** 1 Center of Nanotechnology, King Abdulaziz University, Jeddah 21589, Saudi Arabia; hshabbirhussain@stu.kau.edu.sa (H.H.); nsalah@kau.edu.sa (N.S.); aalshahri@kau.edu.sa (A.A.); amemic@gmail.com (A.M.); moansari@kau.edu.sa (M.O.A.); 2 Physics Department, Faculty of Science, King Abdulaziz University, Jeddah 21589, Saudi Arabia; 3 Functional Materials Group, Applied Physics Department, School of Engineering Sciences, KTH Royal Institute of Technology, AlbaNova Universitetscentrum, 10691 Stockholm, Sweden

**Keywords:** freestanding electrode, activated carbon, carbon nanotubes, capacitive deionization, water desalination

## Abstract

Freshwater reserves are being polluted every day due to the industrial revolution. Man-made activities have adverse effects upon the ecosystem. It is thus the hour of need to explore newer technologies to save and purify water for the growing human population. Capacitive deionization (CDI) is being considered as an emerging technique for removal of excess ions to produce potable water including desalination. Herein, cost-effective activated carbon incorporated with carbon nanotubes (CNT) was used as a freestanding electrode. Further, the desalination efficiency of the designed electrodes was tuned by varying binder concentration, i.e., polyvinylidene difluoride (PVDF) in the activated carbon powder and CNT mixture. PVDF concentration of 5, 7.5, 10, and 12.5 wt% was selected to optimize the freestanding electrode formation and further applied for desalination of water. PVDF content affected the surface morphology, specific surface area, and functional groups of the freestanding electrodes. Moreover, the electrical conductivity and specific surface area changed with PVDF concentration, which ultimately affected the desalination capacity using the freestanding electrodes. This study paves the way to produce cost effective carbon-based freestanding electrodes for capacitive deionization and other applications including battery electrodes.

## 1. Introduction

The demand for drinking water has been continuously increasing over the past few decades, due in part to population increases but also due to the depletion of existing freshwater resources [1]. This is even more exacerbated by the fact that currently less than 3% of freshwater is safe and drinkable. This lack of fresh and drinkable water makes it that much more difficult to meet sustainable development goals especially in countries with limited existing infrastructure. Taken together, it seems that there is a looming fresh and drinkable water crisis on the horizon [2].

To avoid freshwater shortages, alternative approaches for drinkable and irrigation water production have been utilized. Some traditional freshwater generation methods include seawater desalination especially in arid and water scarce areas and climates [3]. There are multiple seawater desalination approaches including electrodialysis (ED), reverse osmosis (RO), and thermal vaporization methods such as multi-stage flash distillation (MST). Today, most water desalination and treatment plants across the globe rely predominately on latter two methods (i.e., RO and MSF [4]). However, RO based processes can be highly cost ineffective and although several factors including production capacity can impact cost, currently it is estimated that for 1 cubic meter of desalinated water, approximately 1.5–2.5 kWh of electricity is required. With current moves toward a sustainable, reduced-carbon society, lower energy consumption for water desalination is needed to significantly decrease the cost of water desalination [5,6].

One recent technology that researchers have been turning to for more energy-efficient water desalination is capacitive deionization (CDI) [7]. The technology is based on removing ions (i.e., salts) from aqueous solutions (i.e., seawater) using electrodes as a key component. Specifically, ion and salt adsorption occurs due to the electric double layer formed around the electrodes when electrical potential is applied and attracts them to the electrode surface interface. After removing the ions, the electrodes can be regenerated by reversing or removing the electrical voltage. In general, CDI requires only 1–1.5 V of electrical potential to operate, thus minimizing potential electrochemical (i.e., oxidation/reduction on electrode surface) reactions. Furthermore, compared to the more traditional desalination methods, CDI has other advantages [8]. For example, CDI does not require high-cost membranes, high pressure pumps, or chemical treatments like other membrane-based technologies. The salt removal can be done at room temperature and pressure with the primary input being a small cell voltage (~1 V) and small electric current source. Together, this makes CDI a much more eco-friendly and sustainable technology for water desalination [9].

Recently, there has been significant progress made in the design and fabrication of CDI electrodes from an array of materials. Many researchers have recently focused on carbon-based materials or precursors while developing the next generation of CDI electrodes. Common carbon-based materials include graphene, carbon nanotubes (CNTs), and carbon aerogels that due to their excellent properties have been used for CDI electrode manufacturing [10]. These electrodes can offer improved performance driven by their high electrical conductivity and controlled pore distribution improving ion adsorption capacity. However, these materials can still be cost prohibitive when developing large scale production of electrodes [11].

One material that could offer very low production cost is activated carbon powder while also providing high specific surface area (1000–2000 m^2^/g). However, one drawback of using activated carbon is that a polymer binder needs to be added during electrode fabrication [12]. This polymer binder addition can lower electrical conductivity of the electrode as resistance builds up due to reduced electrical contact between the carbon particles. To offset this, additional highly conductive carbon materials can be added at low concentrations to improve electrode performance [13]. Furthermore, manufacturing the electrode might require high temperature treatment that could result in poor bonding between different carbon precursors. Therefore, it is critical to optimize and blend the different components during electrode fabrication to achieve best possible results for improving the CDI performances [14].

In this work, we used activated carbon powder and single wall carbon nanotubes (SWCNTs) to fabricate a freestanding CDI electrode, which is cost effective in contrast to the electrodes pasted on expensive supports, i.e., graphite sheets or carbon cloth. We studied how varying the concentration of polyvinylidene fluoride (PVDF) as a polymer binder affects the properties of the electrode. Specifically, we investigated the physicochemical (vibrational spectroscopy and elemental analysis), structural (mechanical and surface morphology), and thermal properties (including thermogravimetric analysis (TGA), and differential scanning calorimetry (DSC)) of the prepared electrodes. Similarly, we studied the effect of binder concentrations on the performance of the fabricated electrodes (i.e., CDI performance, CV profiling). We were able to optimize the binder concentration in order to provide the optimum pore size distribution and specific surface area of the freestanding electrically conducting electrodes for improved deionization performance.

## 2. Materials and Methods

### 2.1. Materials

Activated carbon (Activated charcoal) powder of 100 mesh particle size was supplied by Sigma-Aldrich (Steinheim am Albuch-Germany) with a surface area of around 1100 m^2^/g. Single wall carbon nanotubes (SWCNTs) of high electrical conductivity (>1000 S/cm in their pellet form) were purchased from Ad-Nano Technologies (Shimoga-Karnataka, India). Poly(vinylidene-fluoride) (PVDF) of Mw = 180 kg mol^−1^ was purchased from Sigma-Aldrich and used as received. Dimethyl sulfoxide (DMSO) and highly pure chloroform were purchased from Sigma-Aldrich.

### 2.2. Synthesis of Freestanding Electrodes

**Figure 1:**
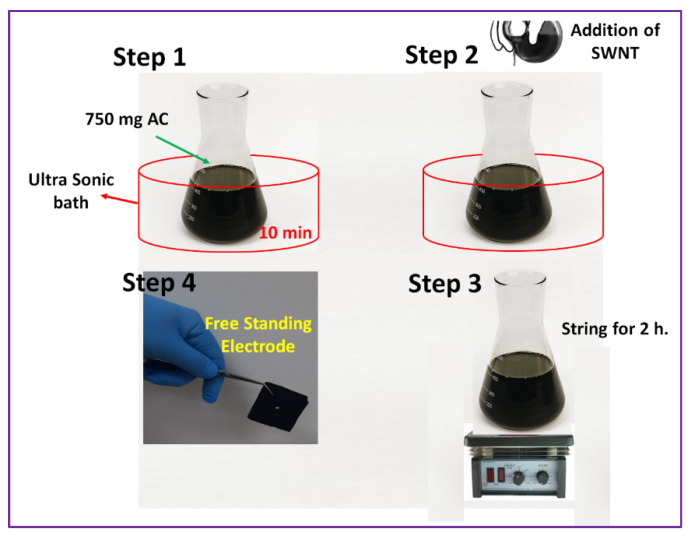
Schematic illustration for the synthesis of freestanding electrode.

For the electrode fabrication, 740 mg of activated carbon was dispersed in 20 mL of DMSO by putting it on an ultrasonic bath for 10 min. To the dispersed activated carbon, PVDF of various concentrations (5, 7.5, 10, and 12.5 wt% of activated carbon) was added and the system was put under vigorous stirring conditions for complete mixing of PVDF with the powder. In another beaker, SWCNT (2 wt% of activated carbon) was dispersed in 10 mL chloroform and subjected to ultrasonic treatment for 10 min. In order to form the slurry, the dispersed SWCNT was added to the PVDF-activated carbon powder mixture and was further stirred for 2 h to obtain a uniform mixture of SWCNT and PVDF-activated carbon. The slurry was drop-casted onto a glass frame (5 × 5 cm) and further dried at 110 °C for 10 s to obtain a well-defined uniform electrode film adhered to the glass surface. Finally, the electrode was peeled off from the glass surface to obtain a freestanding SWCNT-PVDF-activated carbon electrode which was stored in a desiccator for further experiments. The thickness of these electrodes ranged from 0.70 mm to 0.72 mm. The schematic representation of electrode fabrication process is given in Figure 1.

### 2.3. Characterization

The surface morphology of fabricated freestanding electrode was investigated by FESEM JEOL (JSM-7600F) while the elemental compositions were confirmed by EDS (Oxford Instrument-X-Max). Specific surface area and pore size distribution were determined from gas adsorption isotherm measurements using Brunauer–Emmett–Teller (BET) surface area analysis (NOVA 1200e). Fourier transform infrared spectroscopy (FTIR) (Thermo scientific Nicolet iS10) was carried out to confirm the attached functional groups. The CDI performance of the fabricated electrode was examined by employing a customized CDI unit from Stockholmwater Technology (SWT), Sweden. The electrochemical properties of the freestanding electrode were performed by using electrochemical workstation (CH instrument) and the mechanical characteristics were estimated from the nanoindentation (NanoTest Micro-materials Wrexham) tests. The contact angle was estimated through Minder Hightech (SDC-200S).

## 3. Results

### 3.1. Surface Morphology

**Figure 2:**
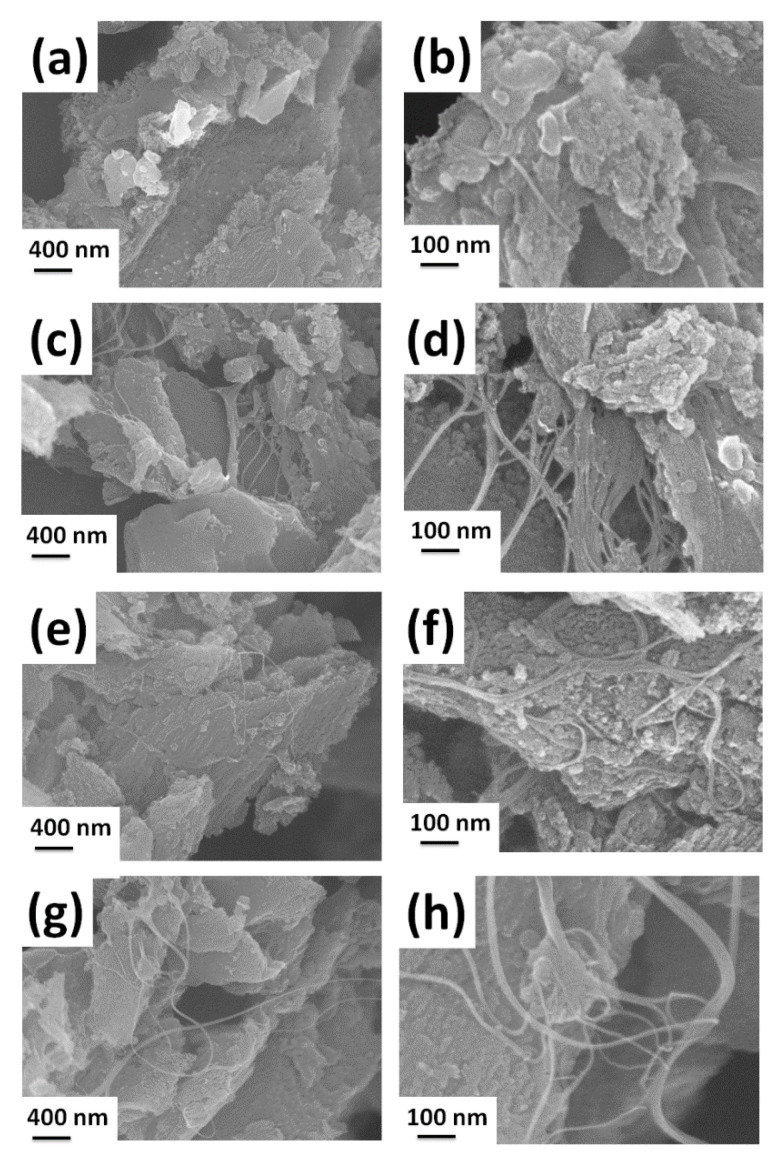
Surface morphology of the fabricated freestanding electrodes with (**a**,**b**) 5%, (**c**,**d**) 7.5%, (**e**,**f**) 10%, and (**g**,**h**) 12.5% of PVDF concentration.

Figure 2a shows the surface characteristics from scanning electron micrographs (SEM) of CNT-AC powder with different weight percentages of PVDF. All the samples seem to be composed of fused irregular flakes, spheres, and agglomerated particles with fine pores, and the bundles of CNTs on the surface as well as partially embedded inside the flakes of AC can be evidently seen. The CNTs of micrometer length can also be seen as a connection between different clusters of flakes and is expected to increase the conductivity along the axial direction while the bundles of well stuck CNTs as observed in Figure 2d can also contribute to the conductivity along the radial direction. It can be seen that with an increase in the PVDF concentration, the stacking of flakes/particles increased, hence an obvious decrease in the porosity. EDS (Table 1 and Supplementary Figure S1) of CNT-AC shows major content of C (from AC and CNTs) and O (due to functionalization of AC and CNTs), while the presence of Na, S, and some other elements might be due to the slight remains of solvent or small amount of impurities from the carbon precursor (Supplementary Figure S2).

### 3.2. Mechanical Property

**Figure 3:**
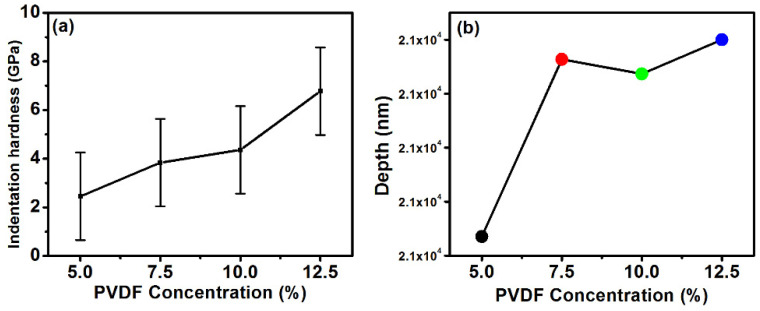
Mechanical characteristic of synthesized electrode at different PVDF concentrations: (**a**) Indentation hardness (**b**) depth penetration.

The mechanical property of the freestanding electrode has a role to play in the CDI fabrication, its performance, and its reliability [15]. Nanoindentation was thus used to test the hardness of the electrode. The results (Figure 3a,b) reveal that there is a change in hardness of electrode with varying PVDF concentration, although the CNT concentration was fixed for all four electrodes. The maximum hardness was 6.77 GPa for electrodes fabricated with 12.5% PVDF (Figure 3a). The PVDF and AC interact through dipole moment and by increasing the PVDF concentration, this dipole moment increases which ultimately enhances the mechanical strength. Moreover, the interaction of PVDF with CNT also affects the strength of electrode [16].

### 3.3. FTIR Results

**Figure 4:**
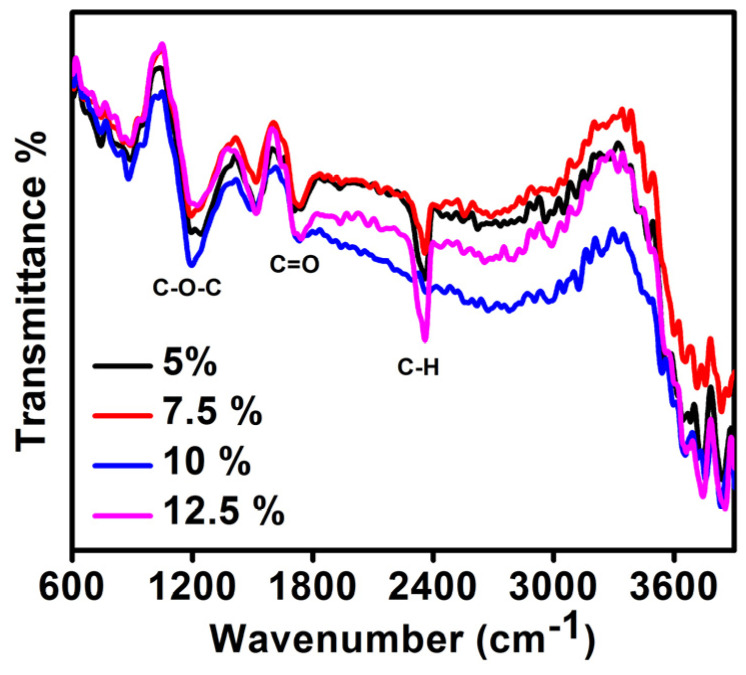
FTIR spectra of synthesized electrodes with varying PVDF concentrations.

**Figure 5:**
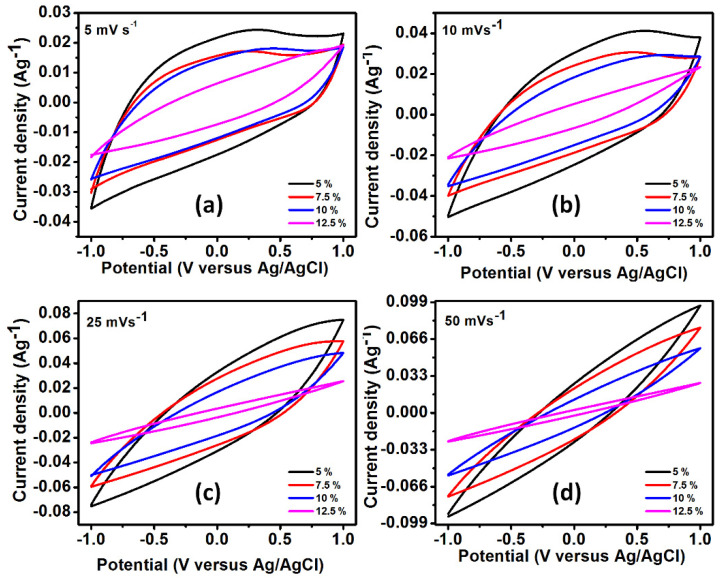
Supercapacitive behavior of synthesized electrode at different PVDF concentration for scan rate (**a**) 5 mV s^−1^, (**b**) 10 mV s^−1^ CV, (**c**) 25 mV s^−1^ CV, and (**d**) 50 mV s^−1^.

FTIR analysis (Figure 4) of the prepared electrode at various percentage of PVDF is presented in Figure 5. The main peaks were detected at 883, 1198, 1490, 1740, 2360, 3660, 3747, and 3856 cm^−1^. The peaks at 883 cm^−1^ correspond to the mixed mode of CH_2_/CF_2_ stretching vibrations and the band at 1198 cm^−1^ is due to the symmetric stretching mode due to CF_2_. The peak at 1490 cm^−1^ and the band contributed by small peaks from 2800 and 3100 cm^−1^ is due to CH_2_, of which the latter is due to the symmetric and asymmetric stretching vibrations of CH_2_ [17,18]. The peak at 1740 cm^−1^ is indicative of the C=O stretching frequency indicating the presence of carboxylic group on carbon during its activation or on the CNT’s surface during its purification while manufacturing. The 2360 cm^−1^ band can be assigned to arise from the CH_2_–CO-group [19]. The absorption peaking around 3500 cm^−1^ is due to the O-H stretching vibrations and the peak at 3747 cm^−1^ is ascribed to the free hydroxyl groups [20]. The FTIR spectra for all weight percentage of PVDF is similar with a slight change in peak intensities is indicative of the fact that PVDF interacts with carbon materials by weak and strong forces.

### 3.4. Electrochemical Characteristics of the Electrodes

**Figure 6:**
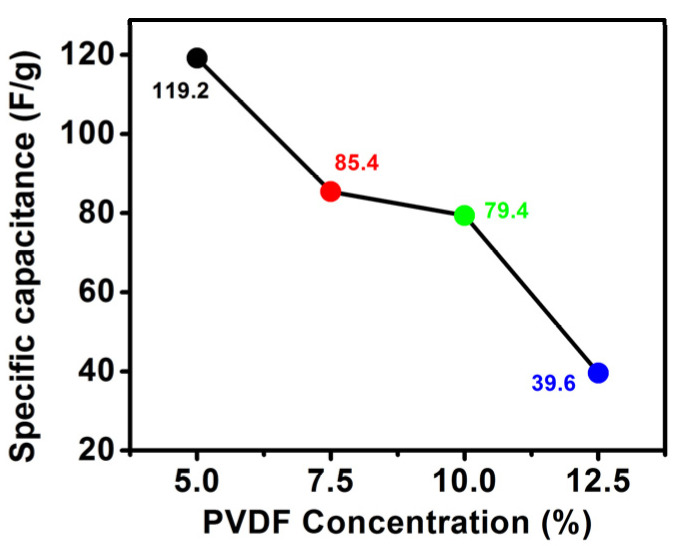
Calculated specific capacitance of electrodes for different PVDF concentration.

The electrochemical analysis of AC-CNT composite with different weight percentage of PVDF was done by cyclic voltammetry (CV). Figure 5a–d shows the CV curves of AC-CNT electrodes at different scan rates of 5, 10, 25, and 50 mV/s in the potential range from −1.0 to 1.0 V. From Figure 6, the observed behavior shows the quasi-rectangular shape which is of a typical electrochemical double layer capacitor and the linear increase with an increase in the current density suggests good electrochemical reversible behavior [21]. With an increase in the scan rate over 5 mV/s, the voltammogram window shifts towards the vertical axis; this might be due to: (i) the decreased pore size and pore volume at higher PVDF concentration, and thus the 12.5% electrode showed poor performance and (ii) the interaction of ions mostly at the outer surface of the electrode and the bulk material inside contributes less to the capacitance due to little interaction.

The specific capacitance of AC-CNT composite was estimated using Equation (1) [22]:

(1) $$C=\frac{\int^{\;}Idv}{2vxm\Delta V}$$

The specific capacitance (Figure 6) of AC-CNT composite was found to be 119.2, 85.4, 79.4, and 39.6 Fg^−1^ at a fixed scan rate of 5 mV/s. The results show that the higher percentage of the PVDF decreases the overall specific capacitance. It might be noticed that the accessible surface area is the key factor regarding the capacitance. With an increase in the PVDF concentration, the overall pore volume and pore size decrease; however, an abrupt increase was seen for 10% PVDF loading. The decrease in pore size and pore volume lowers the electrochemical performance by decreasing the surface-active sites responsible for the conduction of electrons during the electrochemical process. Apart from this, the lower conductivity of PVDF is also responsible for the decrease in the electrochemical performance at higher loading. The electronic conductivity (in-plane and through-plane) of the prepared freestanding electrode was determined through Linseis (HCS-10). Conductivity of in-plane and through-plane was found to be almost the same which is attributed to the isotropic nature. The conductivity of electrodes at 5% of PVDF was 130 s/m, which further reduced with an increase in the PVDF concentration. Therefore, the electrodes prepared at PVDF concentration of 7.5%, 10%, and 12.5% have conductivity of, respectively, 121, 116, and 103 s/m.

### 3.5. Surface Area Analysis

**Figure 7:**
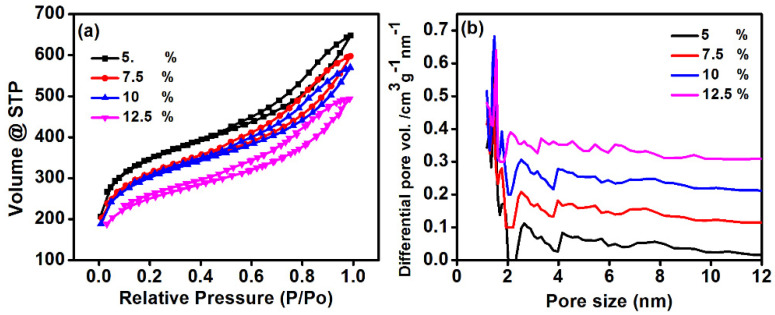
Brunauer–Emmett–Teller (BET) (**a**) isotherm and (**b**) pore size analysis of perpend elected at various PVDF concentration.

Specific surface area of the electrode has a great impact on the CDI performance. The pore size, pore volume, and average diameter of the material are important as it assists to absorb the ions during the CDI process [23]. Therefore, Brunauer–Emmett–Teller (BET) analysis of prepared electrodes was performed to estimate the pore size distribution and specific surface area for different PVDF concentrations. The N_2_ adsorption–desorption isotherm of commercial activated carbon is presented in Figure S3 and the surface area analysis of commercial activated carbon is given in Table S1. Figure 7 shows the N_2_ adsorption–desorption isotherms of the prepared electrodes that conform to Type-IV isotherm. The The results revealed the change in the surface area and pore size distribution which also showed the influence of PVDF concentration during the electrode preparation. The specific surface area and pore size at PVDF 7.5% were found to be approximately 721 m^2^g^−1^ and 3.26 nm, respectively. However, a change in surface area and pore size was noticed while changing the PVDF concentration from 7.5 to 12.5%. The increased concentration of PVDF leads to enhancing the viscosity of the solution while preparing the electrode which could ultimately increase the mass transfer resistance [24] between the activated carbon, SWNT, and DMSO. This eventually leads to slighter higher surface area and pore sizes in the electrode (Table 1). Therefore, it can be concluded that PVDF is also a major factor to change the surface area which ultimately affects the CDI performance of the electrodes. The calculated BET parameters such as specific surface area, pore volume, and total volume at various concentrations of PVDF are shown in Table 2.

### 3.6. Deionization of Saline Water

**Figure 8:**
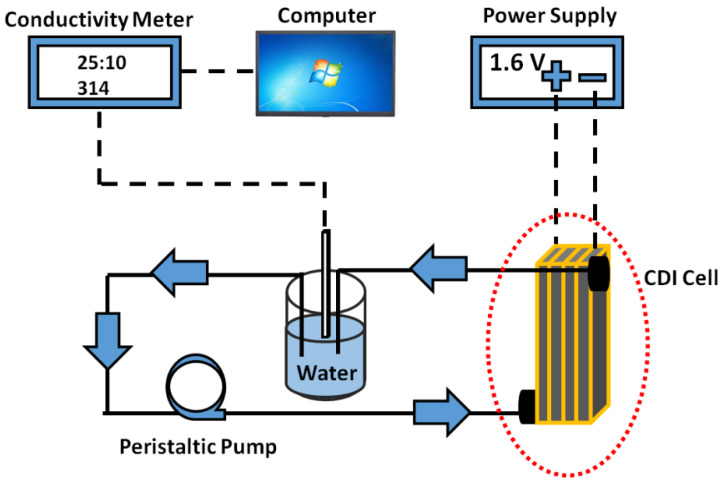
The schematic illustration to evaluate the desalination efficiency using the fabricated electrodes containing various PVDF concentrations.

The CDI electrodes were fixed in the device and continuous conductivity measurements were recorded at room temperature (~25 °C). Water flow rate was controlled by a peristaltic pump and was fixed for all experiments at 6 mL/min. A solution of NaCl (1000 ppm) was used to evaluate the salt removal efficiency of prepared electrodes with various PVDF (5, 7.5, 10, and 12.5%) concentrations. An applied voltage of 1.6 V was fixed for all testing the device performances with the four prepared electrodes. However, before applying the voltage, the NaCl water solution was passed through the CDI cell to attain the equilibrium. Afterwards, salt removal efficiency, salt absorption, average salt absorption, and deionization capacity of the prepared electrodes was calculated. A schematic diagram of the experimental setup for CDI is shown in Figure 8.

**Figure 9:**
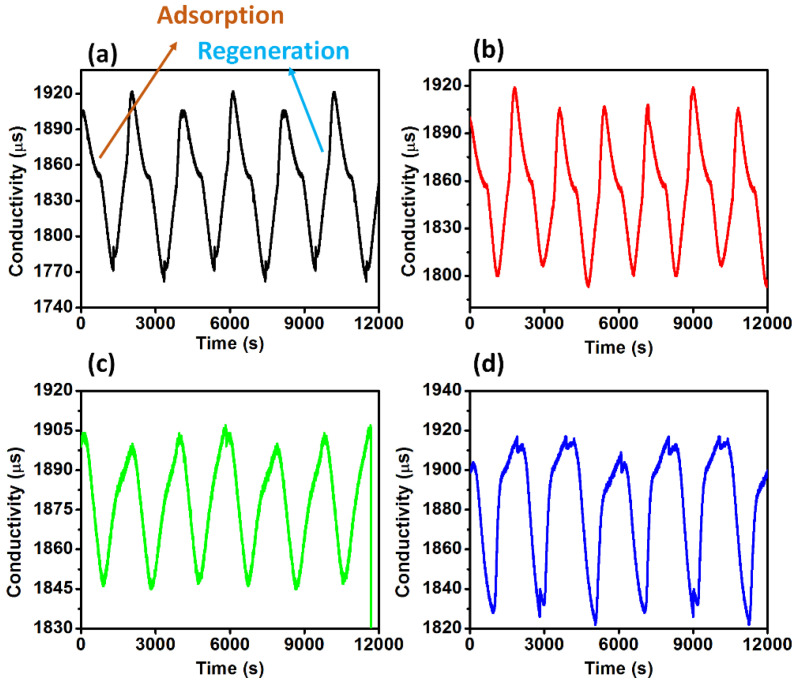
The change in conductivity of the NaCl water solution during the cyclic absorption and regeneration for the prepared electrode at PVDF concentration (**a**) 5%, (**b**) 7.5%, (**c**) 10%, and (**d**) 12.5%.

The cyclic change in the conductivity of NaCl water solution by using prepared electrode for adsorption and desorption is shown in Figure 9a–d. The absorption cycle shows a decrease in the initial conductivity of NaCl water solution which is attributed to the capture of Na^+^ and Cl^−^ ions from NaCl water solution. During the regeneration cycle the electrode starts releasing the Na^+^ and Cl^−^ ions into water which ultimately start increasing the conductivity to the initial conductivity. However, during this cyclic process some loss in the conductivity was absorbed, which indicates trapping of some Na^+^ and Cl^−^ ions on the electrodes. However, changes in the cyclic behavior were noticed in Figure 9a–d, which indicated the change in CDI performance of electrodes by varying the PVDF concentration.

The salt absorption capacity of the prepared electrode was estimated by the following Equation [25].

(2) $$\mathrm{Salt}\;\mathrm{adsorption}\;\mathrm{capacity}\;\left(\frac{\mathrm{mg}}{g}\right)=\frac{(C_{o}-C_{f})V}{m}$$

**Figure 10:**
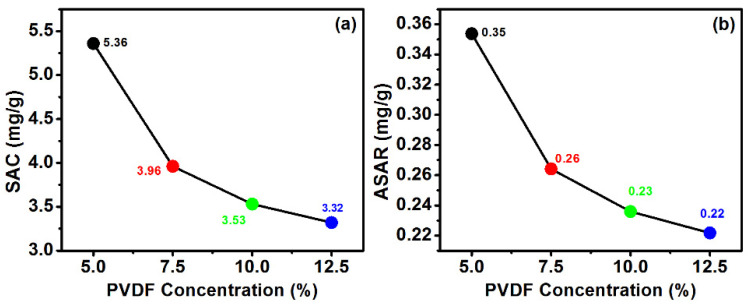
(**a**) Salt absorption and (**b**) average salt absorption capacity of the prepared electrode.

In the above equation, Co is the initial salt concentration while C_f_ is the final salt concentration with the time interval while m is the mass. Figure 10 shows the salt absorption capacity of the prepared electrode at various PVDF concentration. The results revealed the change in salt absorption while changing the PVDF concentration from 5 to 12.5%. The maximum salt absorption was 5.30 mg/g for electrode prepared with 5% of PVDF. However, salt absorption capacity for 7.5, 10, and 12.5% was respectively 3.96, 3.53, and 3.32 mg/g. This variation in the salt absorption capacity further leads to changes in the average salt absorption capacity of the prepared electrode and was calculated by the following relation [26].

(3) $$\mathrm{ASAR}\;\left(\frac{\mathrm{mg}}{g}\right)=\frac{\mathrm{SAC}}{t}$$

where *t* is the time while SAC is the total salt absorption capacity in a specific interval of time.

### 3.7. Contact Angle Measurement

**Figure 11:**
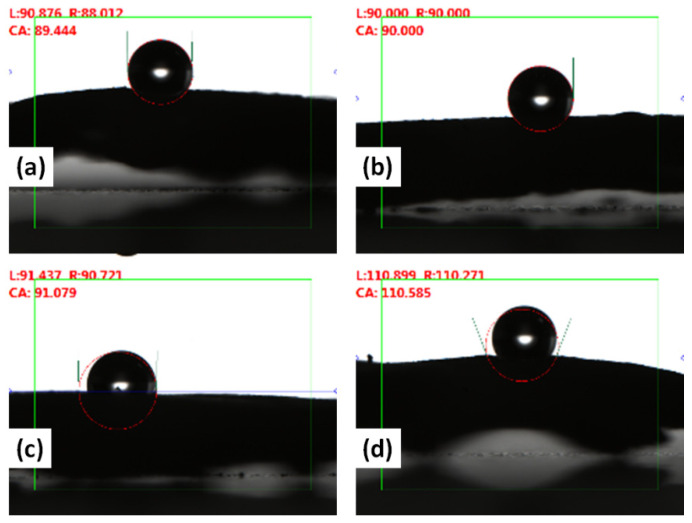
Contact angle for the prepared electrode at PVDF concentration (**a**) 5%, (**b**) 7.5%, (**c**) 10%, and (**d**) 12.5%.

The contact angle of fabricated freestanding electrodes (Figure 11) was investigated to find the hydrophilicity or hydrophobicity. A drop of 1.00 μL was fixed for all the electrodes. The mean contact angle reading for electrodes at 5% PVDF was noticed to be 89.4°. However, the contact angle increased to 110.58° with the increase in PVDF from 5 to 12.5%. This increasing trend in the contact angle with the rise in PVDF concentration is attributed to the hydrophobic nature of PVDF [27]. Moreover, pore size, surface roughness, and surface tension also affected the contact angle of the fabricated freestanding electrodes [28].

## 4. Conclusions

In summary, freestanding electrodes of activated carbon and CNTs with varying percentages of binder, namely PVDF, were fabricated. Morphological analysis showed dispersed CNTs inside the activated carbon with CNTs deeply embedded as well as on the surface. The FTIR analysis with varying PVDF content showed changes in peak position and intensity which suggests interaction of PVDF with the AC and CNTs by weak and strong forces. The mechanical testing by nanoindentation showed maximum hardness of 6.77 GPa for 7.5% of PVDF content in the electrode which also confirmed interaction of PVDF with CNT and activated carbon, and this ultimately affects the strength of electrode. The CV results showed typical electrochemical double layer capacitor and the electrode with 5% of PVDF showed maximum specific capacitance of 119.2 Fg^−1^ at the fixed scan rate of 5 mV/s. The CDI performance of all the electrodes showed adsorption of Na^+^ and Cl^−^ and desorption during regeneration cycle observed by decrease and increase in the conductivity, respectively. Of all the electrodes, the maximum salt absorption was 5.30 mg/g for the 5% electrode.

## Figures and Tables

**Table 1:** Energy-dispersive spectroscopy (EDS) elemental composition of the fabricated freestanding electrodes at various PVDF concentration.

PVDF Concentration (%)	C_k_ (Atomic %)	F_k_ (Atomic %)
5	83.53	7.96
7.5	74.79	14.25
10	78.95	7.15
12.5	74.31	12.96

**Table 2:** Surface area analysis from BET.

Sample (PVDF)	S_BET_ (m^2^g^−1^)	V_total_ (cm^3^g^−1^)	V_micro_ (cm^3^g^−1^)	pore_ave_ (nm)	V_total_ (cm^3^g^−1^)
5%	721	0.51	0.70	3.26	0.92
7.5%	521	0.50	0.64	3.24	0.85
10%	233	0.46	0.62	3.05	0.81
12.5%	110	0.43	0.48	3.01	0.64

## Data Availability

The data will be made available upon request.

## Supplementary Materials

The following supporting information can be downloaded at: https://www.mdpi.com/article/10.3390/polym14142891/s1, Figure S1: EDS elemental composition of the fabricated freestanding electrodes (a) 5%, (b) 7.5%, (c) 10%, and (d) 12.5% of PVDF concentration, Figure S2: EDS analysis of carbon precursor used to prepare the freestanding electrode, Figure S3: BET analysis of commercial activated carbon used to prepare the free standing electrode, Table S1: Surface area analysis of commercial activated carbon used to prepare the free standing electrode.
