# Supplementary material for: Freestanding Activated Carbon Nanocomposite Electrodes for Capacitive Deionization of Water
Source: Polymers (Basel). 2022 Jul 16;14(14):2891. doi: 10.3390/polym14142891 (PMC9319057; doi:10.3390/polym14142891)
Supplement: Supplementary file 1 [file polymers-14-02891-s001.zip › polymers-1771526-supplementary.pdf]

**Supplementary:**

# Free standing Activated Carbon nanocomposite electrodes for Capacitive Deionization of water

**Humair Hussain,[1,2] Asim Jilani,[1] Numan Salah,[1] Ahmed Alshahrie,[1,2] Adnan Memic,[1] Mohammad Omaish Ansari,[1] Joydeep Dutta[1,3]**

[1] Center of Nanotechnology, King Abdulaziz University, Jeddah 21589, Saudi Arabia.

[2] Physics Department, Faculty of Science, King Abdulaziz University, Jeddah 21589, Saudi Arabia.

[3] Functional Materials Group, Applied Physics Department, School of Engineering Sciences, KTH Royal Institute of Technology, AlbaNova universitetscentrum, 106 91 Stockholm, Sweden.

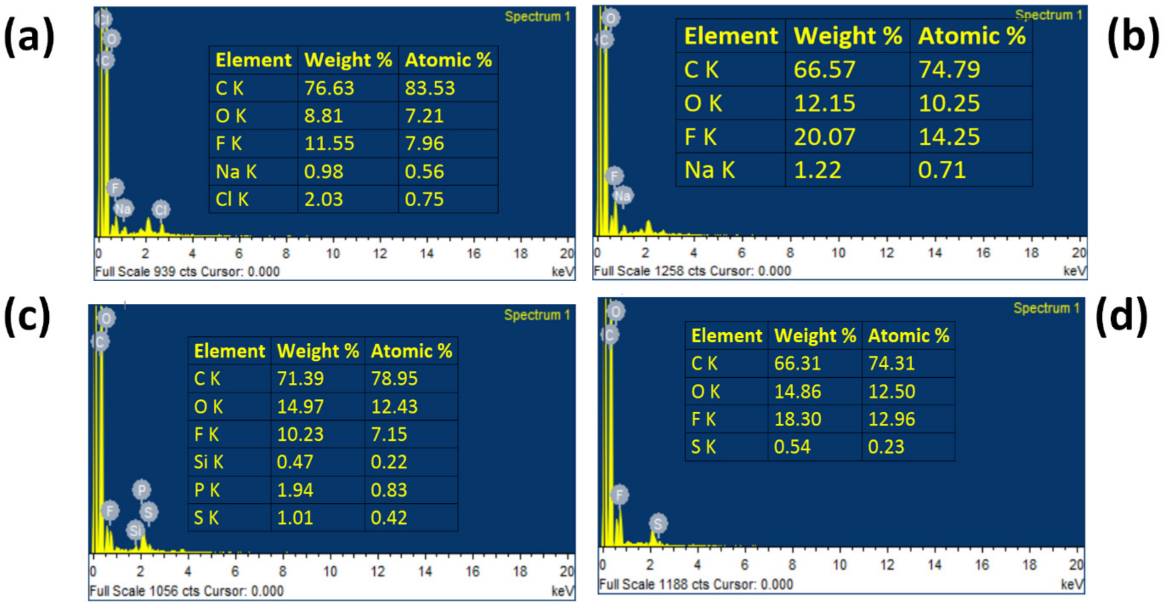


**Figure S1:** EDS elemental composition of the fabricated free standing electrodes (a) 5%, (b) 7.5%, (c) 10% and (d) 12.5% of PVDF concentration.

**Texture Properties of commercial activated carbon**

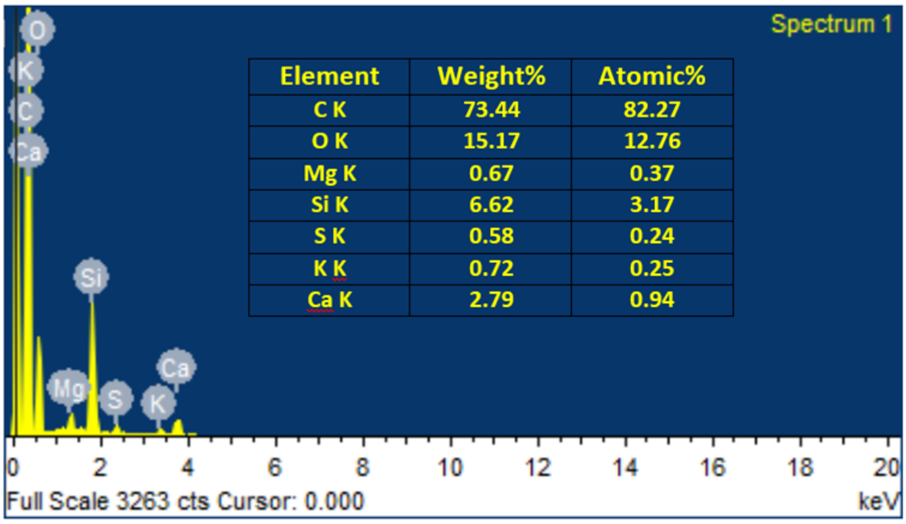


**Figure S2:** EDS analysis of commercial activated carbon used to prepare the free standing electrode.

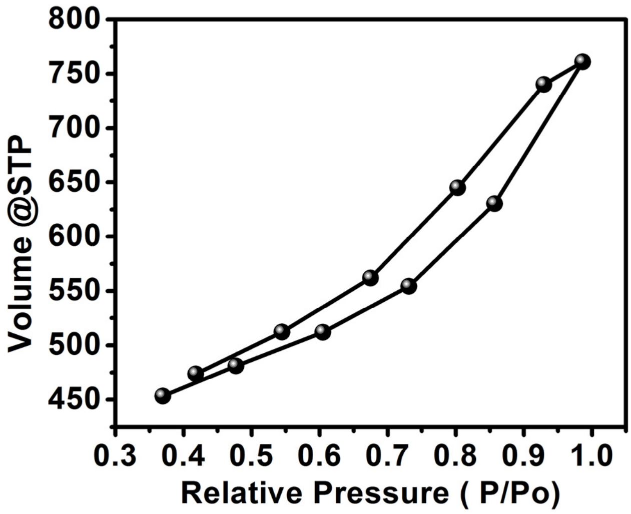


**Figure S3:** BET analysis of commercial activated carbon used to prepare the free standing electrode.

**Table S1**: Surface area analysis of commercial activated carbon used to prepare the free standing electrode.

| SBET (m2g-1) | Vtotal (cm3g-1) | Vmicro (cm3g-1) | poreave (nm) | Vtotal (cm3g-1) |
|---|---|---|---|---|
| 1250 | 0.53 | 0.78 | 3.33 | 0.98 |
